# BIOLEACH: A New Decision Support Model for the Real-Time Management of Municipal Solid Waste Bioreactor Landfills

**DOI:** 10.3390/ijerph17051675

**Published:** 2020-03-04

**Authors:** Javier Rodrigo-Ilarri, María-Elena Rodrigo-Clavero, Eduardo Cassiraga

**Affiliations:** Instituto de Ingeniería del Agua y Medio Ambiente (IIAMA), Universitat Politècnica de València, 46022 Valencia, Spain; marodcla@upv.es (M.-E.R.-C.); efc@dihma.upv.es (E.C.)

**Keywords:** landfill, leachate, bioreactor, biogas, modeling, simulation, municipal solid waste

## Abstract

This paper introduces BIOLEACH, a new decision support model for the real-time management of municipal solid waste bioreactor landfills that allows estimating the leachate and biogas production. Leachate production is estimated using an adaptation of the water balance equation which considers every hydrological component and the water consumed by anaerobic organic matter degradation to create biogas and the leachate recirculation flows pumped from the landfill pond under a bioreactor management scheme. Landfill gas production is estimated considering the leachate formation process as a coupled effect through the production or consumption of water. BIOLEACH uses waste production and climate data at monthly scale and computes leachate production accounting for the actual conditions inside the waste mass. Biogas production is computed simultaneously, considering the available water to adjust the chemical organic matter biodegradation. BIOLEACH is a valuable bioreactor managing tool as it allows calculating the recirculation volume of leachate that ensures optimal moisture conditions inside the waste mass and therefore maximizing biogas production. As an illustrative example of a BIOLEACH application, the model has been applied to a real landfill located in Murcia Region (Spain) showing the economic and environmental benefits derived from leachate superficial recirculation.

## 1. Introduction

The term municipal solid waste (MSW) covers all kind of wastes generated in a community, including those generated by municipal services and treatment plants and excluding those coming from industrial and agricultural processes [1]. Landfilling is currently the most common engineering approach to managing MSW rejections and the environmental control of the landfill is a critical issue to guarantee ecosystem equilibrium and environmental protection [2,3]. Recent approaches to implementing new technologies focus on recycling, re-use, and thermal and energetic valorization of waste. These technologies include composting, biomethanization, incineration techniques with energy re-use and biofuel production. However, only composting appears to have had a practical implementation at field scale. Following the Spanish National Waste Plan [4], 44% of the total annual MSW generation in Spain is still managed through landfill deposition.

Unfortunately, the use of simulation software as a tool for the design, operation and monitoring of landfills is not as widespread in the field of municipal solid waste landfills as in other environmental engineering fields such as wastewater treatment plants [5]. However, biogas and leachate production are two of the most relevant environmental issues to address during both the operational and post-closure phases of an MSW landfill [6,7].

Leachate production is the final result of a hydrological process related with the infiltration of precipitation through the MSW mass forming a complex organic liquid. This fluid shows the characteristics of a wastewater concentrate and may induce extremely negative effects on surface water and groundwater quality if it is released into the environment [8]. Leachate contains soluble organic, inorganic, bacteriological constituents and suspended solids [9]. From an economical perspective, leachate treatment costs are one of the most expensive items to be considered in the daily operation of a landfill facility. Leachate management operations usually include both the removal of the leachate from the pond where it is stored and its transportation to an external wastewater treatment plant, which may be located at a long distance from the landfill.

Landfill gas (biogas) mainly consists of methane (45% to 60% by volume), biogenic carbon dioxide (40% to 55%) and other greenhouse gases in minor proportions. The biogas formation process derives from the anaerobic decomposition of the organic matter of the MSW stored in the landfill [10,11]. If a correct collection of this biogas is not carried out, combustion or even spontaneous explosions may occur inside the waste mass, compromising the stability of the landfill slopes and the health of the facility’s workers.

A bioreactor landfill is defined as a sanitary landfill site that uses enhanced microbiological processes to transform and stabilize the decomposable organic waste constituents [12]. Common bioreactor control processes focus on the analysis of the leachate recirculation, the waste mass evolution and the efficiency of the biogas production process. Operating the landfill as a bioreactor ensures an environmentally sustainable management minimizing environmental impacts and making landfill operations economically more profitable. Some reasons are generally cited to justify the bioreactor technology [13,14]: (i) rapid organic waste conversion/stabilization, (ii) maximizing of landfill gas capture for energy recovery projects, (iii) increased landfill space capacity due to rapid settlement during operational time period, (iv) improved leachate treatment and storage, and (v) reduction in post-closure care.

Bioreactor landfills allow for enhanced leachate recirculation and faster solid waste degradation. This is achieved adding supplemental water to the waste and/or recirculating leachate [15,16,17]. Some common methods for leachate recirculation are vertical wells [18,19,20,21], horizontal trenches [22,23,24,25] and drainage blankets [26,27]

Understanding the characteristics of landfill leachate is essential to managing it in the most efficient manner [28]. However, improvement of methane generation for gas collection and sale from landfills is hampered by a general lack in understanding of landfill processes at field-scale [29].

## 2. Scope and Objectives

A literature review has been done to identify available models able to predict landfill gas production. Two of the most cited ones are LandGEM [30] and GasSim [31]. Despite they compute biogas production, they are not designed as real-time support models to provide practical information to the user about specific managing strategies that may finally increase gas production. HELP [32] is a widely used leachate production model. MODUELO 4.0 [33] and LAST [34] are computer tools to simulate hydrological, biodegradation and settlement processes inside the landfill. MODUELO is one of the most complete tools for modeling water balance in landfills while LAST is a recent model that reduces the amount of necessary data to perform simulations. Both models estimate, among other parameters, biogas moisture content and chemical composition. The majority of numerical tools for landfill management simulation focus on environmental risk objectives in accordance with environmental regulations [35].

This work introduces BIOLEACH, a new decision support model for the real-time management of municipal solid waste bioreactor landfills that estimates leachate and biogas productions. Table 1 shows a comparison of the main features of these available models.

BIOLEACH overcomes some limitations of HELP model: (i) BIOLEACH better reproduces the progressive filling of the landfill volume over time, (ii) BIOLEACH includes some key processes that affect leachate production such as water transfer between different levels due to waste compression and consolidation, and water consumption due to waste biodegradation.

LAST does not consider bioreactor modeling through leachate recirculation, this being one of BIOLEACH main features.

MODUELO is a complex model that includes all the features considered in BIOLEACH. The key difference between them lies in the model structure and the numerical formulations used internally in the model to simulate the different processes. As shown in Section 3, BIOLEACH uses relatively simple empirical and analytical equations so the number of necessary input data are significantly reduced. On the contrary, MODUELO simulations require many different input parameters that must be specified by the user before running the model. Usually, most of them may not be available or may be completely unknown due to the lack of specific information about the landfill. Some of the most important parameters to perform MODUELO simulations are [5,33]: (i) Horton model parameters, (ii) minimum and maximum infiltration rates, (iii) vertical and horizontal waste hydraulic conductivities, (iv) values of waste drainage, moisture and porosity, (v) field capacity, (vi) wilting point, (vii) hydraulic conductivity variations with depth and (viii) different organic matter degradation rates related with acetogenesis, methanogenesis and hydrolysis processes. As it will be explained below, BIOLEACH uses a simpler approximation that needs a reduced number of parameters.

BIOLEACH has been designed as a completely new decision support tool [36] to provide information and support landfill operators so leachate recirculation volumes are justified in order to maximize the monthly biogas production while minimizing monthly leachate volumes to be managed by external water treatment facilities. The model wills to fulfill needs of the landfill managers, addressing for a provided specific MSW chemical composition and under site-specific meteorological conditions. Chemical characterization of MSW and maximum leachate storage capacity of the pond must also be known. 

Once the specific information is available, BIOLEACH addresses questions such as: (i) what is the monthly expected leachate production? (ii) what is the actual monthly production of biogas? (iii) how can biogas production be maximized by optimally using the leachate volume stored inside the pond? and (iv) if leachate recirculation is convenient, how should such volumes be distributed inside the MSW mass in order to maximize efficiency in terms of biogas production?

The relevance of the above issues is critical both from the environmental and economic point of view. This is due to the fact that environmental risks and economic costs associated with leachate management on external facilities are key aspects during landfill management operations.

## 3. Materials and Methods

### 3.1. Conceptual Model

BIOLEACH is a new mathematical model that allows evaluating the joint production of leachate and biogas at a monthly scale through the application of water balance techniques.

**Figure 1:**
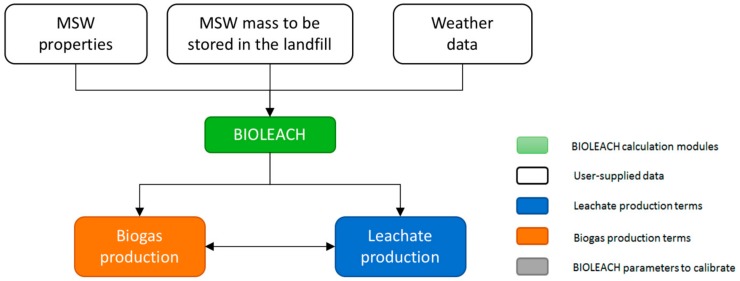
Flowchart of the conceptual scheme implemented in BIOLEACH.

Furthermore, BIOLEACH is designed so leachate and biogas formation processes are considered in a coupled way. It provides a mathematical management tool that considers bioreactor management of an MSW landfill, thus trying to respond in the best possible way to the physic-chemical-biological processes that actually develop inside the MSW mass. The model inputs which must be provided by the user are: (i) the MSW chemical characterization, (ii) the monthly mass of MSW to be stored in the landfill and (iii) the local weather data obtained from the local weather station (Figure 1).

Based on this information, BIOLEACH computes biogas (CH_4_ + CO_2_) and leachate monthly volume productions and compares the actual monthly production of biogas with the maximum biogas production that would be obtained under optimal conditions for these specific MSW characteristics for the same month. Leachate production is computed accounting for the specific local weather conditions at the landfill site.

**Figure 2:**
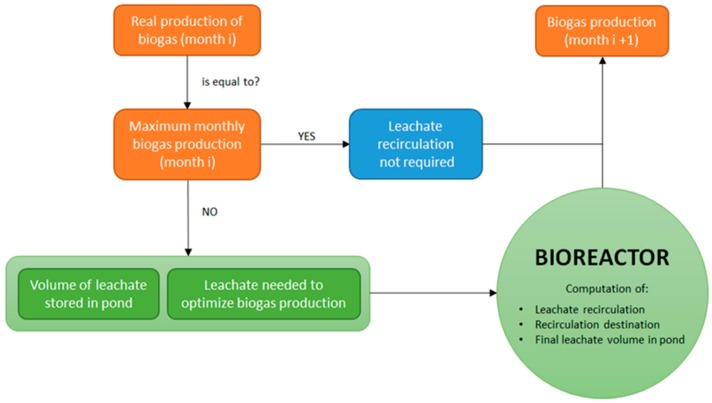
Flowchart of the conceptual scheme implemented in BIOLEACH.

Using an adaptation of the water balance equation and accounting for the MSW water storage capacities, the model internally evaluates the water volumes vertically transferred between the horizontal landfill layers also accounting for leachate recirculation volumes that may be pumped from the leachate pond back to the landfill. Water balance calculations integrate those performed by the biogas module to evaluate water consumption and water vapor generation during the biogas formation process. The flowchart of the conceptual model to simulate the behavior of the bioreactor landfill is shown in Figure 2.

### 3.2. The Water Balance Equation to Compute Leachate Production

Every month the model compares actual biogas production (which is either measured by the landfill operator or theoretically computed by the model) with the maximum biogas production obtained by the model under optimal conditions. If these two values are equal, no leachate recirculation is needed as waste moisture content is optimum. However, if actual biogas production is lower than the maximum one, the model calculates the leachate recirculation volume comparing the actual leachate volume stored in the pond with the leachate volume needed to maximize biogas production following the methodology described below. The decision about the need for leachate recirculation is done by the bioreactor module. Once the recirculation volume has been defined, the bioreactor module also establishes the optimal depth inside the MSW mass where recirculated leachate should be injected as well as the final leachate volume stored in the pond.

**Figure 3:**
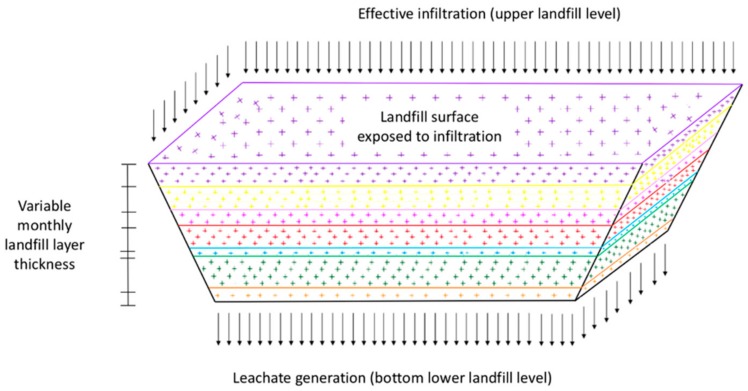
Spatial discretization of the landfill volume.

Calculations performed by the bioreactor module to compute leachate volumes are written in terms of water balance equations. The bioreactor volume is conceptualized by a set of horizontal layers with variable thickness and variable superficial area so effective infiltration values on the upper landfill level provide different water inputs every month. Figure 3 shows the scheme of the spatial discretization considered by the model.

On each layer, the water balance equation is computed monthly as shown in Equation (1):

ΔS_MSW_ = W_MSW_ + W_MC_ + W_UP_ + W_R_ − W_B_ − W_VA_ − W_LO_ (1)

where ΔS_MSW_ (kg of water)/(m^3^ of MSW per month) is the water storage variation inside the MSW mass. The water inputs terms are:

- W_MSW_: MSW moisture content. This value depends on the MSW characterization tests. Typically, moisture content will be higher when MSW comes from a house-to-house system and lower when they are rejections from an MSW treatment plant
- W_MC_: Covering material moisture content
- W_UP_: Water transfer from the immediately upper layer. On the higher layer of the landfill this amount is equal to the effective infiltration rate (precipitation minus evapotranspiration)
- W_R_: Leachate volume recirculated from the pond when the landfill is operated as a bioreactor. Making this parameter equal to zero allows performing calculations for traditional landfill management techniques

Besides, the water outputs terms are:

- W_B_: Water consumed on the biogas formation process, computed using the anaerobic biodegradation of organic matter formulation explained below
- W_VA_: Generation of water vapor during the biogas formation process, computed using the ideal gas equation, pV = nRT (where p: absolute gas pressure (Pa), V: volume (m^3^), n: moles of gas, R: universal constant of ideal gases = 8.314472 J/(K/mol), T: absolute temperature (K))
- W_LO_: Water transfer to the bottom layer. This amount is equal to the leachate production on the lower layer of the landfill. This volume is stored inside the leachate pond at the end of the month

**Figure 4:**
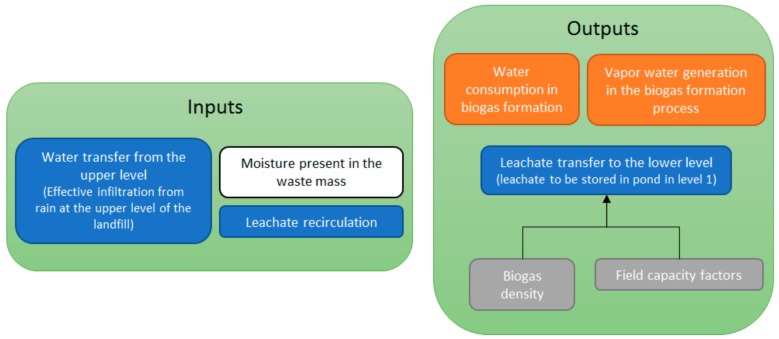
Balance equation terms considered by the model.

Figure 4 shows the balance equation terms considered by the model.

BIOLEACH evaluates leachate production considering that the leachate collection system is fully efficient and fully operational throughout the entire lifetime of the facility. This leachate collection system ensures that leachate is transferred to an onsite storage pond.

To perform all the water balance calculations, the following two parameters should also be calibrated: (i) biogas density (kg/m^3^), which usually varies between 1.1–1.6 kg/m^3^ and (ii) MSW field capacity (FC), evaluated as shown in Equation (2) [37,38]:

FC = A − B × (W/(C + W))(2)

where A, B and C are three coefficients to calibrate and W is the total weight of the specific layer.

Moisture content on the MSW mass is computed from the solid waste characterization provided by the user. If the landfill is already in operation, geophysical methods could be applied to estimate these parameters [39].

### 3.3. The Biogas Production Module

After the MSW characterization, the total weight of each waste component, their water content and the total mass of C, H, O, N included on the organic fraction of the solid waste are determined. BIOLEACH considers both the rapidly (RDW) and slowly (SDW) decomposable fractions of the MSW, as well as the non-decomposable fraction of waste (NDW). Following [1], Equation (3) shows the stoichiometric formulation that allows estimating the biogas production under optimal conditions (total organic matter biodegradation) in terms of the MSW chemical composition and neglecting the effects of sulfur under anaerobic conditions.

(3) $$C_{a}H_{b}O_{c}N_{d}+\left((,\frac{4a-b-2c+3d}{4},)\right)H_{2}O\to \left((,\frac{4a+b-2c-3d}{4},)\right){\mathrm{CH}}_{4}+\left((,\frac{4a-b+2c+3d}{4},)\right){\mathrm{CO}}_{2}+{\mathrm{dNH}}_{3}$$

The term C_a_H_b_O_c_N_d_ represents (on a molar basis) the composition of the organic matter at the start of the process. Coefficients a, b, c and d are the molar proportions of carbon, hydrogen, oxygen and nitrogen respectively. These coefficients are calibrated by BIOLEACH for a given MSW to be stored in the landfill. Coefficients depend on the proportions of the different components of the MSW. This methodology assumes that the biodegradable portion of the organic waste is fully stabilized, and it will finally degrade in methane, CO_2_ and ammonia. The velocity of this conversion depends on the content of RDW and SDW inside the MSW mass and water availability.

**Figure 5:**
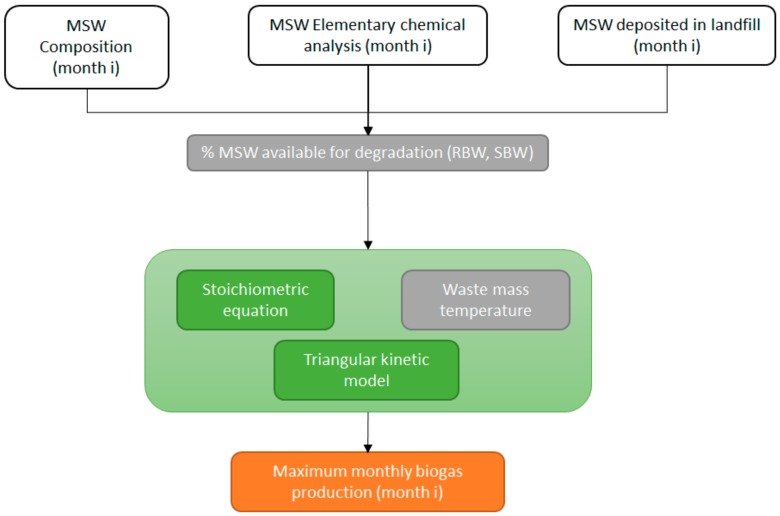
Biogas production module flowchart.

The biogas production module flowchart implemented in BIOLEACH (Figure 5) computes the maximum monthly biogas production independently for RDW and SDW. BIOLEACH considers a triangular kinetic model for the anaerobic biodegradation process. The user must calibrate the percentage of MSW that is actually available for degradation and specify the patterns of the triangular model: (i) total biodegradation time for RDW and SDW and (ii) time for maximum degradation ratio for RDW and SDW.

### 3.4. The Bioreactor Module

**Figure 6:**
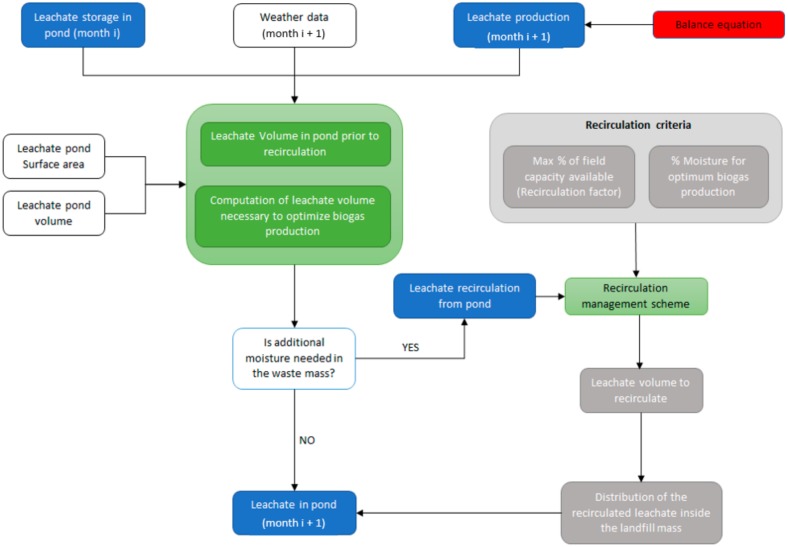
Bioreactor module flowchart.

The core of the BIOLEACH model is the bioreactor module whose flowchart is shown in Figure 6. The user-specified information refers to the monthly weather data, the landfill surface area available for infiltration and the volume and surface of the leachate pond. Based on all this previous information, calculations are internally computed by the numerical model ensuring that the water balance equation is always fulfilled. The model compares the water content inside the MSW mass at every layer and informs the user about the maximum volume of leachate that could be recirculated back inside the landfill volume at the end of every month.

Once the maximum monthly biogas production has been calculated based on all the available data, BIOLEACH allows simulating the monthly operation of the landfill by calculating the monthly leachate production and comparing the monthly biogas production with the maximum theoretical production considering the triangular kinetic model explained in Section 3.3.

The conceptual model implemented in BIOLEACH considers that the deviations in the production of biogas with respect to the maximum production are due to the absence of optimal moisture content conditions in the waste mass. BIOLEACH keeps track of the leachate volume stored in the pond every month and calculates the leachate volume existing in the following month (considering or not leachate recirculation into the landfill) according to the consumption of water in the biogas formation process, the external water inputs by infiltration and the MSW moisture content.

Besides, the user must provide as input the surface of the leachate pond and its maximum storage capacity, as well as the local weather data at monthly scale (precipitation and evaporation). The surface of the leachate pond is necessary to assess the loss of leachate by evaporation in the pond, as well as to compute the increase of leachate volume by direct precipitation on it. The maximum storage volume of the pond is used to inform the user about possible overflows.

To account for the effect of abnormally high precipitation events in relation to the annual average precipitation value ($P_{\mathrm{av}}$) a High Precipitation Factor (HPF) is defined. If monthly precipitation is higher than ($\mathrm{HPF}\cdot P_{\mathrm{av}}$) then all this monthly precipitation infiltrates without accounting for any evaporation effects. HPF allows considering the effects of high return period precipitation events common in Mediterranean basins.

### 3.5. Recirculation Criteria

After the monthly biogas production calculations have been performed, the comparison with the optimal biogas production may justify the necessity to provide additional moisture content in the waste mass. In such a case, the model suggests as an intermediate result the possibility of a leachate recirculation based on two different criteria that must be defined a priori by the user and can be modified during the modeling process: (i) Criterion 1: verify that MSW moisture content guarantees optimal biogas production and (ii) Criterion 2: verify that MSW moisture content is equal to a certain value related with the MSW field capacity. Results provided by BIOLEACH depend on the definition of these two criteria.

Regarding criterion 1, it has been seen that the biogas production process is carried out when the MSW moisture content is in the range between 40% and 70%. [40,41]. Lower values of water content inhibit the process of bacterial degradation of organic matter, while higher values may compromise the stability of the waste mass and inhibiting the biogas formation process. These facts are controlled by defining a factor (α) as the target water content inside MSW. Therefore, criterion 1 can be written in terms of α to maximize biogas production as shown in Equation (4).

(4) $$W_{\mathrm{RL}}=\frac{\alpha }{1-\alpha }\cdot W_{\mathrm{MSW},\mathrm{dry}}\;\;0\;<\;\alpha \;<\;1$$

where W_RL_ is the recirculated leachate weight and W_MSW,dry_ is the dry weight of MSW.

Criterion 2 is considered in BIOLEACH to ensure the existence of a sufficiently high level of moisture content inside the MSW mass but avoiding saturation conditions. The model allows that water content after leachate recirculation is up to 30% greater than field capacity (FC). This is done defining a factor (β) that affects field capacity. Therefore, criterion 2 can be written in terms of the target water content inside MSW over field capacity as shown in Equation (5).

(5) $$W_{\mathrm{RL}}=\frac{\beta \cdot \mathrm{FC}}{1-\beta \cdot \mathrm{FC}}\cdot W_{\mathrm{MSW},\mathrm{dry}}\;\;1\;<\;\beta \;<\;1.3$$

Once these two recirculation criteria have been defined, BIOLEACH suggests to the operator the possibility of performing leachate recirculation actions at specific levels inside the bioreactor landfill. The model considers that it is possible to perform leachate recirculation in horizontal infiltration ditches built for this purpose once a year (every twelve-monthly levels). These ditches should be perfectly functional during the landfill operational life (therefore during the operation, closure, environmental restoration and post-closure phases). Recirculation suggestions provided by BIOLEACH are designed to maintain a homogeneously distributed moisture content inside the mass of waste, taking into account the occurrence of localized rain events that could increase the moisture content in the higher levels. In such a case, the model would suggest recirculating leachate volumes to lower levels of the bioreactor landfill.

Nevertheless, decision about the acceptance or not of the recirculation suggestions provided by BIOLEACH must be taken by the landfill operator, who will finally manually control both the leachate volume actually recirculated and the infiltration ditch(es) to which redirect such volume(s).

A classic landfill management simulation would be carried out if leachate recirculation volumes remain null in time. In such a case, the entire leachate production should be managed externally.

### 3.6. Numerical Formulation

The numerical formulation and implementation of the conceptual model explained above has been programmed in Visual Basic as an Excel spreadsheet in three independent modules: (i) Module 1-Maximum monthly biogas production calculation module, (ii) Module 2-Bioreactor management module and (iii) Module 3-Operation rules module.

A typical execution of the BIOLEACH model begins with the execution of Module 1 to calculate the maximum monthly biogas production. To run Module 1 it is necessary to input the MSW monthly composition, the MSW elementary chemical analysis as well as the amount of MSW deposited monthly in the landfill. Table 2 shows the characterization data of the MSW of Murcia Region landfill (Spain) that was used on the model application explained below.

Based on the previous data and the temperature value of the waste mass, which must be specified by the user and which is considered constant in the simulation, BIOLEACH internally performs the calculations to estimate the biogas production per unit of MSW mass (m^3^ biogas/kg MSW) independently for the RDW and SDW fractions. The amount of water vapor produced in the anaerobic decomposition reaction of organic matter, the amount of water consumed in the process and the water consumed on the biogas formation are also calculated. Module 1 is completed with the implementation of the kinetic model for RDW and SDW fractions independently considering the fractions of waste that are actually available for degradation calibrated by the user.

By default, the triangular kinetic model implemented in the model considers a 15-year degradation of the SDW. Therefore, for a 10-years operational period, biogas production extends during 25 years after the beginning of the landfill operations and reaches its maximum value by the end of year 10 when the RDW stored in years 1 to 5 have fully degraded.

Module 2-Bioreactor management module computes monthly leachate production and makes the recirculation suggestions, integrating the calculations performed by Module 1 in the internal calculations of the bioreactor management module. This management module is designed to be used during the operation of the bioreactor landfill and has been programmed in a spreadsheet consisting of five interrelated blocks that describe all the characteristics of the landfill for each layer in each month: (i) Block 1: General characteristics of each MSW layer, (ii) Block 2: Water balance calculations at the current layer, (iii) Block 3: Control of the volume of leachate stored in the pond before recirculation, (iv) Block 4: Recirculation criteria and (v) Block 5: Control of the volume of leachate stored in the pond after recirculation.

Table 3 shows the full list of model parameters that are included to perform the simulations on each one of the five blocks.

Module 3-Operation rules module uses those results obtained from the execution of Module 2 to compute leachate volumes that could be recirculated inside each one of the available infiltration ditches inside the landfill in order to guarantee optimal moisture content conditions to maximize monthly biogas production. Module 3 compares these leachate volumes and the actual volumes stored in the leachate pond and suggests to the user, where appropriate, to recirculate all or part of this leachate volume stored in the pond and conveniently distribute it between the existing infiltration ditches. This process is called “recirculation suggestions”. The criterion used by BIOLEACH to determine the recirculation suggestions between the different ditches is to obtain optimal moisture conditions inside the entire mass of waste, taking into account the following four considerations: (i) the moisture content actually existing in each layer, (ii) the occurrence of specific precipitation events that could saturate the upper levels of the landfill, (iii) the MSW age, which defines the humidity needs according to the chosen kinetics model, and (iv) the necessity of ensuring waste mass stability complying with the restrictions imposed by the previously defined recirculation criteria.

Actual recirculation on each layer must be finally set by the model user considering the recirculation suggestions provided by BIOLEACH but also accounting for the real situation of the landfill daily operation and the state of the bioreactor. The model checks whether the needed volume of leachate to recirculate does actually exist in the pond or if it is higher, informing the user in each case.

In addition to these three modules, BIOLEACH provides graphical information and tables that allow analyzing how the fundamental parameters that define the bioreactor management process evolve with time. The graphic information generated by the model refers to: (i) temporal evolution of the recirculation criteria, (ii) temporal evolution of biogas production compared to optimal production, (iii) temporal evolution of the volume of leachate stored in the pond, and (iv) temporal evolution of the volume of recirculated leachate.

## 4. Case Study: MSW Landfill Management in Murcia Region (Spain)

To demonstrate the use of BIOLEACH on a real case study, the model has been applied to a new MSW landfill facility located in Murcia Region (Spain). Simulations were designed to consider two different management scenarios:

(i) Scenario 1: operating the facility as a classic landfill without leachate recirculation
(ii) Scenario 2: operating the landfill as a bioreactor allowing for leachate recirculation to the landfill surface.

### 4.1. Available Data

The Murcia Region landfill started operations in January 2019 and it stores the waste rejections of the MSW composting plant located near the landfill site. The composting plant provides waste management service to a group of eight different municipalities with a total population of 246,823 (2018 census data).

The main characteristics of the solid waste managing system are: (i) the initial annual production of MSW stored in the landfill is 66,132 t/year; (ii) MSW production increases at a linear annual rate equal to 0.8%; (iii) landfill capacity provides 10 years of operation and (iv) annual MSW mass is evenly distributed in 12 horizontal layers each one corresponding to the monthly inputs. The landfill facility includes a leachate storage pool with a total capacity of 4500 m^3^.

MSW total mass is formed by three different fractions: the rapidly decomposable waste (RDW), the slowly decomposable waste (SDW) and the non-decomposable waste (NDW). A triangular kinetic model for the biogas formation has been adopted. Under this assumption, RDW fraction degrades completely in 5 years while SDW degrades completely in 15 years. Maximum degradation rates are found 1 and 5 years after the MSW has been stored in the landfill, respectively. The stoichiometric formulation for the complete biodegradation of organic matter shown in Equation (3) has been used.

**Figure 7:**
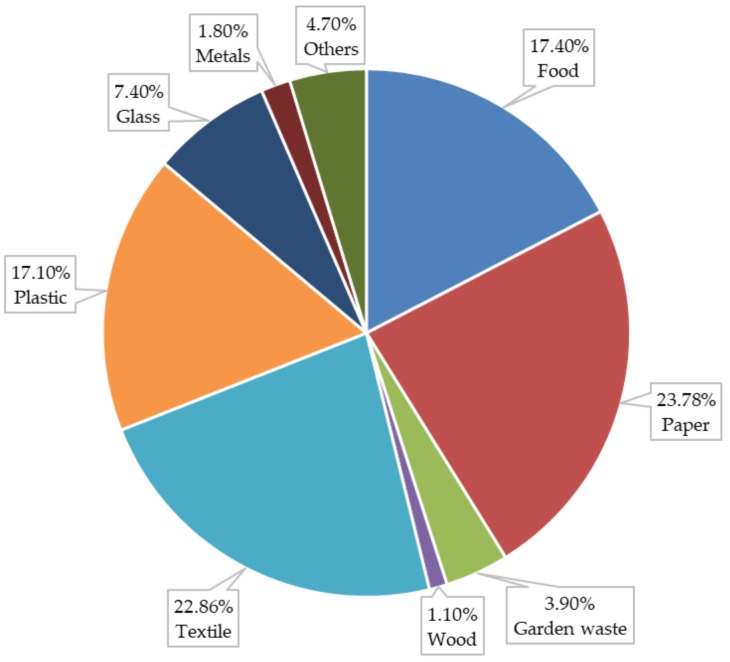
Landfill MSW characterization.

A detailed MSW characterization and chemical elemental analysis of the MSW are those shown in Table 1. Figure 7 graphically shows the landfill MSW characterization. MSW include a very high content of textile (22.86%) and paper (23.78%) while food and plastic waste are found in lower proportions (17.40% and 17.10%, respectively).

**Figure 8:**
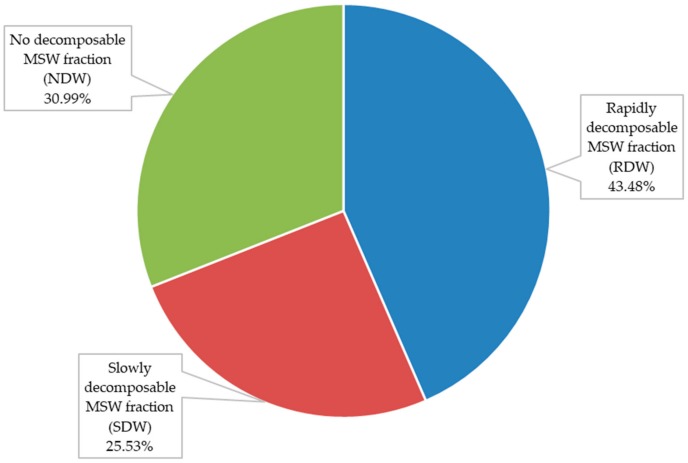
Biodegradability potential of MSW stored at the landfill site.

Figure 8 shows the biodegradability potential of each MSW fraction. Rapidly decomposable MSW fraction (RDW) is 43.48% of total MSW, while the slowly decomposable fraction (SDW) is 25.53%. No decomposable MSW fraction (NDW) is 30.99% of the total MSW.

The anaerobic biodegradation of the organic fraction for RDW and SDW are described by Equations (6) and (7), respectively. These stoichiometric reactions were obtained using the total organic matter biodegradation model described in Section 3.3.

(6) $$C_{47}H_{75}O_{33}N+12H_{2}O\to 24{\mathrm{CH}}_{4}+22{\mathrm{CO}}_{2}+{\mathrm{NH}}_{3}$$

(7) $$C_{14}H_{21}O_{6}N+7H_{2}O\to 8{\mathrm{CH}}_{4}+7{\mathrm{CO}}_{2}+{\mathrm{NH}}_{3}$$

**Figure 9:**
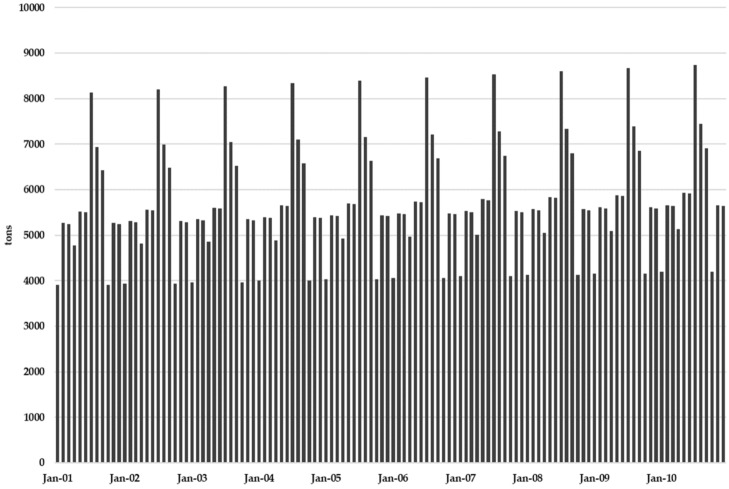
Monthly landfill MSW production.

MSW landfill inputs for the period January to September 2019 is available. Starting with these available data, Figure 9 shows the monthly MSW inputs stored in the landfill for a 10-year simulation period considering that MSW production increases at a linear annual rate of 0.8%.

**Figure 10:**
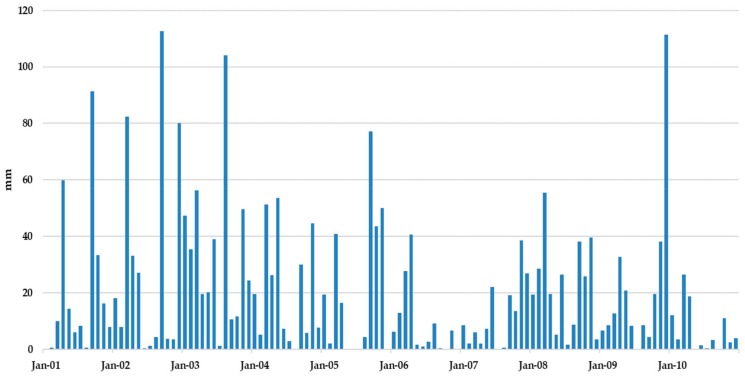
Precipitation data for a 10-year simulation period.

Monthly precipitation data were obtained from the landfill local weather station using (i) the values actually recorded from January to November 2019 for these months and (ii) the precipitation values recorded from 2008 to 2017 in the following months to complete a 10 years simulation period (120 months). Figure 10 shows a graphical representation of the precipitation data. Low rainfall and a very high evaporation capacity were observed at the site, being these values typical from a Mediterranean basin. High return period precipitation events are eventually found during the month of September and are easily identified as peaks of the precipitation graph.

### 4.2. Model Calibration

The model calibration process ensures that the model parameters are set to reproduce the actual landfill operation state. No real biogas production data are available. However, monthly landfill leachate production is known. A total volume of 2794 m^3^ of leachate was produced during the first nine months of operation of the landfill. Leachate production values are therefore considered as targets for the model calibration.

The calibration process was done following an iterative method to determine: (i) the proportions of RDW and SDW that are actually available for degradation and (ii) the best value of parameters α and β and (iii) the value of parameter HPF.

Calculations showed that only 50% of RDW and 30% of SDW were available for degradation, α=0.4, β=1.3 and HPF=2.0.

**Figure 11:**
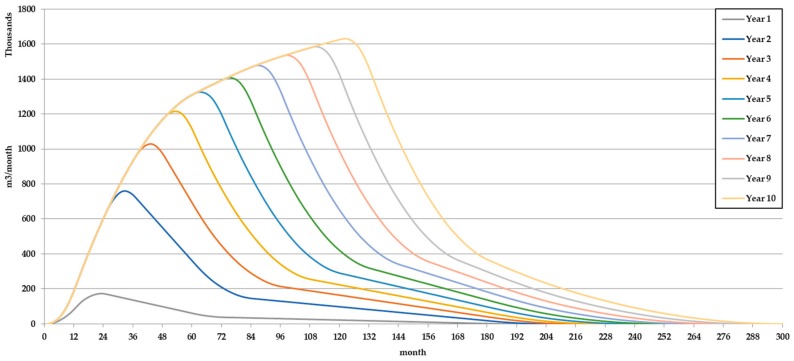
Maximum monthly biogas production rate for each 1–10 year.

**Figure 12:**
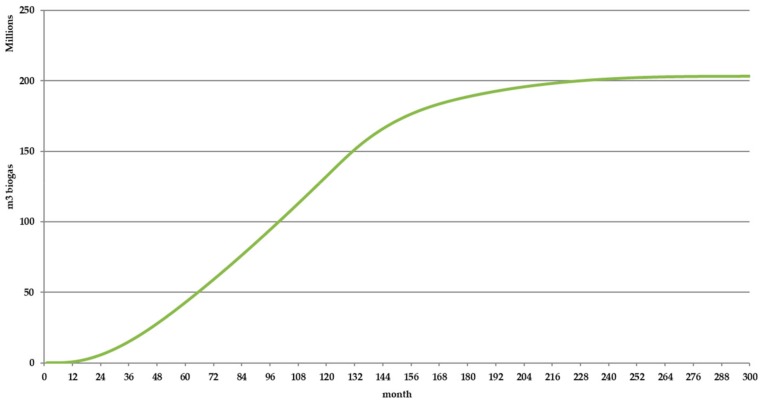
Accumulated biogas production under optimal conditions.

Using these values inside BIOLEACH Module 1-Maximum monthly biogas production calculation module, the optimal biogas production for the specific MSW stored in the landfill was found. Figure 11 shows the maximum monthly biogas generation rate for each one of the 10 years included in the simulation period. Figure 12 shows the accumulated biogas production under optimal conditions.

**Figure 13:**
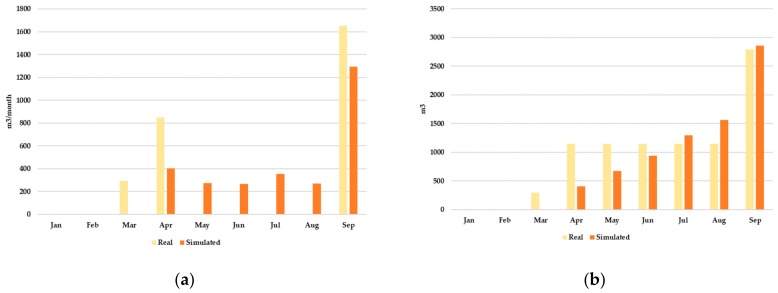
Comparison of actual and simulated monthly leachate productions during the first nine months. (**a**) Monthly leachate production; (**b**) Accumulated leachate production.

The model calibration was checked comparing the actual and simulated monthly leachate productions (Figure 13). Simulations lead to excellent results when comparing total leachate production during the first nine months of landfill operation (2794 m^3^) and the corresponding simulated value (2858 m^3^), showing that the model was able to predict leachate production after the parameter calibration process was finished.

### 4.3. Model Results

As it was said above, to evaluate the benefits of a bioreactor management scheme in comparison with classic landfill management techniques, two different scenarios were considered: (i) Scenario 1: operating the facility as a classic landfill without leachate recirculation and (ii) Scenario 2: operating the landfill as a bioreactor allowing for leachate recirculation to the landfill surface.

#### 4.3.1. Scenario 1—Classic Landfill Management Scheme

**Figure 14:**
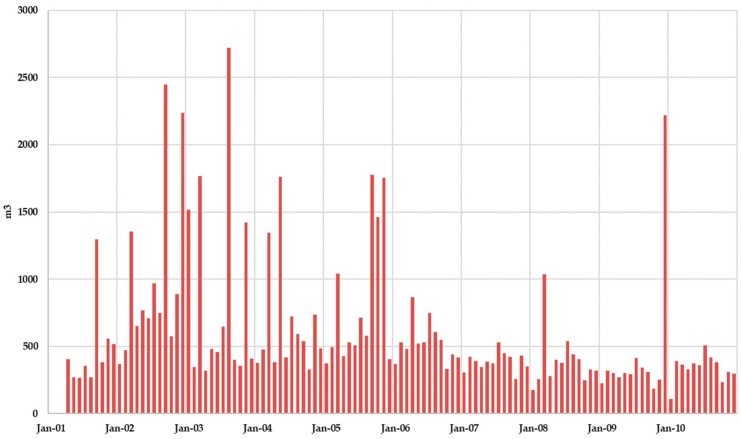
Scenario 1. Monthly leachate production.

Figure 14 shows the monthly leachate generation without superficial leachate recirculation. It was observed that leachate production exceeded 2000 m^3^/month only in four months. When comparing leachate production with effective infiltration rate (precipitation minus evaporation), peak values of the leachate production series coincide with peak values of the effective infiltration series, although reciprocally it is not always true, showing that depending on the MSW moisture content in the landfill its response to a rain event in terms of leachate production may be more or less visible.

**Figure 15:**
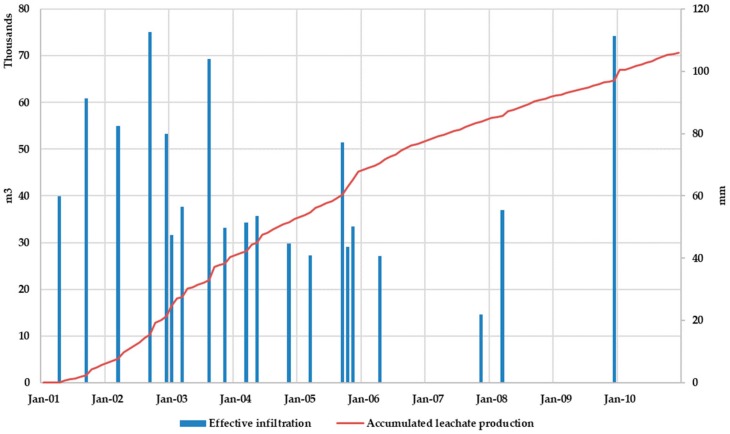
Scenario 1. Effective infiltration and accumulated leachate production.

Figure 15 shows the comparison between effective infiltration rate and the accumulated leachate production during the simulation period. Results show that, after a precipitation event, the slope in the accumulated leachate production curve increases so the model reflects the observed physical reality.

During the analysis it was admitted the following management criterion: if leachate volume stored in the pond exceeded 3000 m^3^ a certain leachate volume would be transferred to an external water treatment plant, so the final leachate volume stored in the pond was 1000 m^3^.

**Figure 16:**
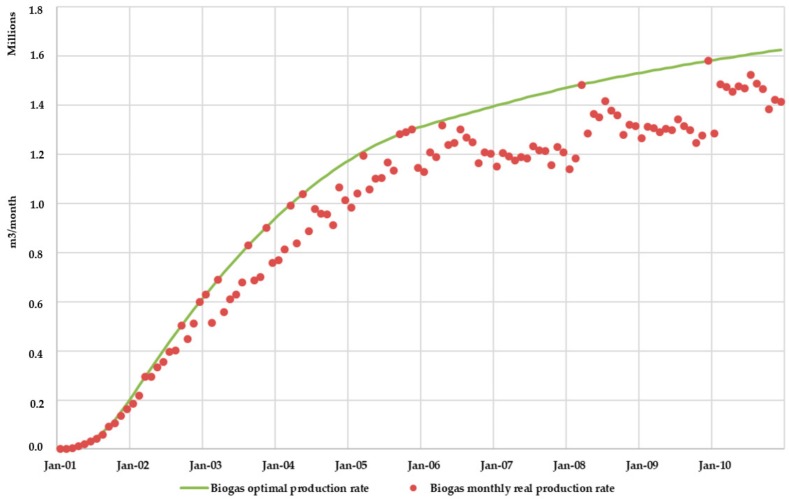
Scenario 1. Biogas monthly real production rate vs optimal production rate.

Concerning biogas production, BIOLEACH model obtained the comparison between the actual monthly biogas generation rates and the optimal production rates (Figure 16). As expected, it was observed that actual biogas production rates are lower than those rates obtained under optimal conditions.

#### 4.3.2. Scenario 2—Bioreactor Landfill Management Scheme

**Figure 17:**
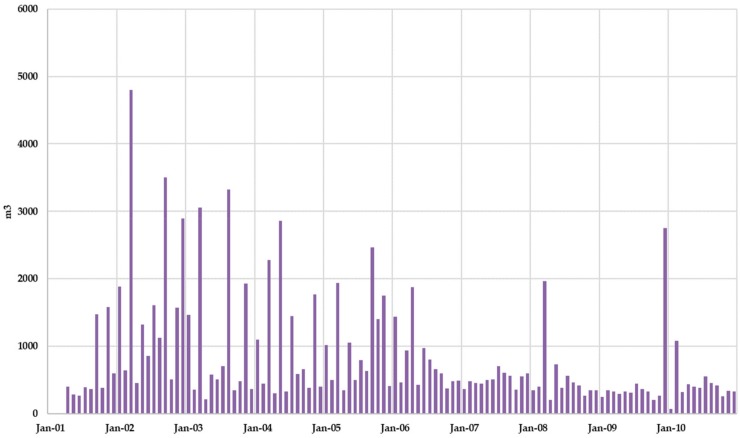
Scenario 2. Monthly leachate production.

Figure 17 shows the evolution of the monthly leachate generation when leachate recirculation is admitted. Leachate recirculation effects are clearly identified comparing Figure 14 and Figure 17. While results found for Scenario 1 (Figure 14) showed that leachate production exceeded 2000 m^3^ only in four months in Scenario 2 this effect was found in nine months.

Results obtained for Scenario 2 when comparing leachate production with effective infiltration rate (precipitation minus evaporation) are similar to those obtained for Scenario 1. Peak values of the leachate production series coincide with peak values of the effective infiltration series, although reciprocally it is not always true, showing that depending on the MSW moisture content in the landfill its response to a rain event in terms of leachate production may be more or less visible.

**Figure 18:**
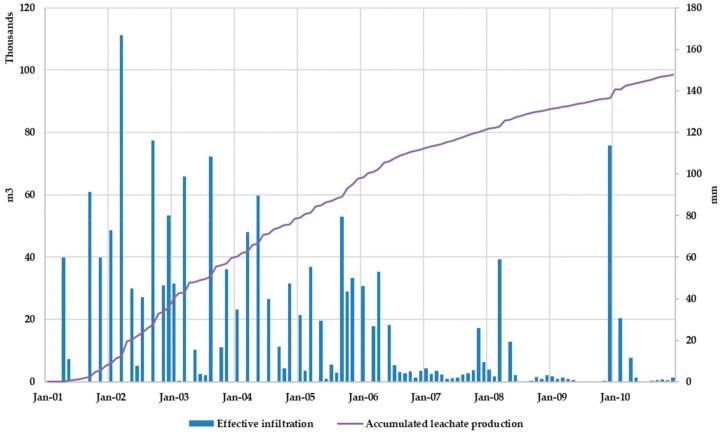
Scenario 2. Effective infiltration and accumulated leachate production.

Figure 18 shows the comparison between effective infiltration rate and accumulated leachate volume production. After a precipitation event, the slope in the accumulated leachate production curve increases, which allows us to state that the model adequately reflects the observed physical reality.

**Figure 19:**
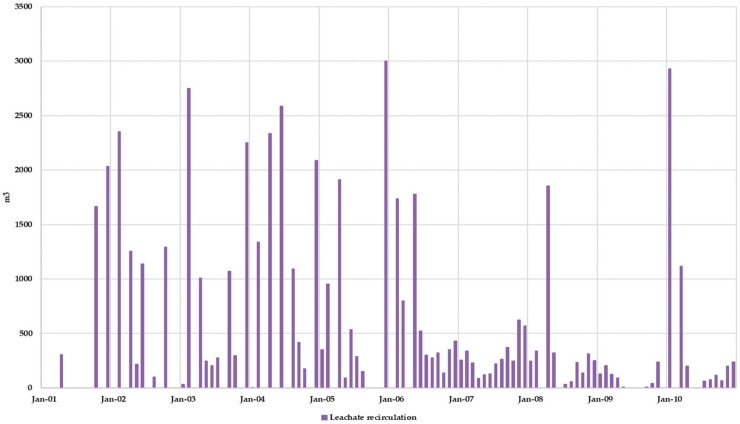
Scenario 2. Monthly volume of recirculated leachate.

Figure 19 shows the monthly volume of recirculated leachate. This volume of recirculated leachate allows drastically reducing the volume of leachate stored in the pool. Besides, the leachate volume to be managed externally is greatly reduced, therefore allowing for a drastic reduction in the external leachate management economic cost. An estimation of this benefit can be done considering an approximate leachate external management cost of 60 €/m^3^. While for Scenario 1 (without considering surface leachate recirculation) this cost was estimated to be 2,600,000 € during the 10-year period, for Scenario 2 (considering surface leachate recirculation) this cost is reduced to 1,100,000 €. This is one of the main benefits of the bioreactor management scheme.

**Figure 20:**
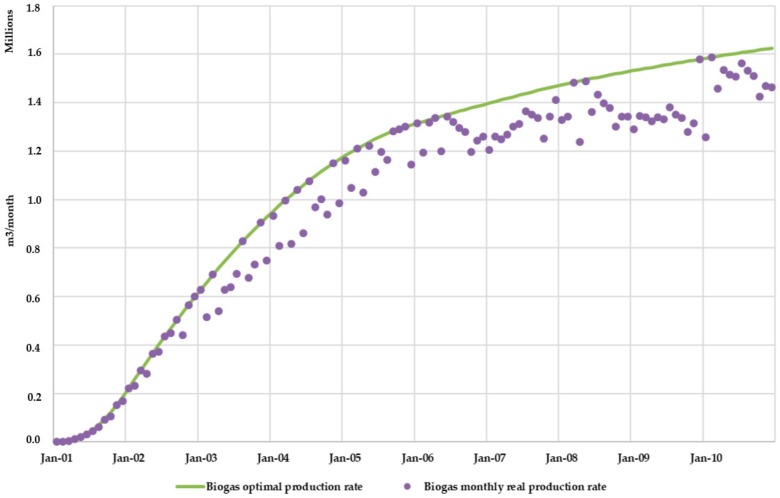
Scenario 2. Biogas monthly real production rate vs optimal production rate.

Figure 20 shows the comparison between the actual monthly biogas generation rates and the optimal production rates for Scenario 2. Once again, as found for Scenario 1, it was observed that actual biogas production rates are lower than those rates obtained under optimal conditions. Additionally, the model allows calculating the volume of water consumed in the biogas formation process and the volume of water vapor generated.

## 5. Discussion

**Figure 21:**
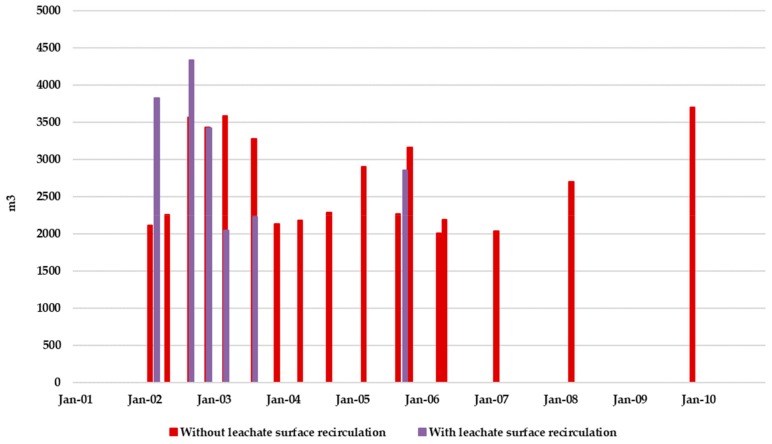
Leachate volumes transferred to external facilities.

**Figure 22:**
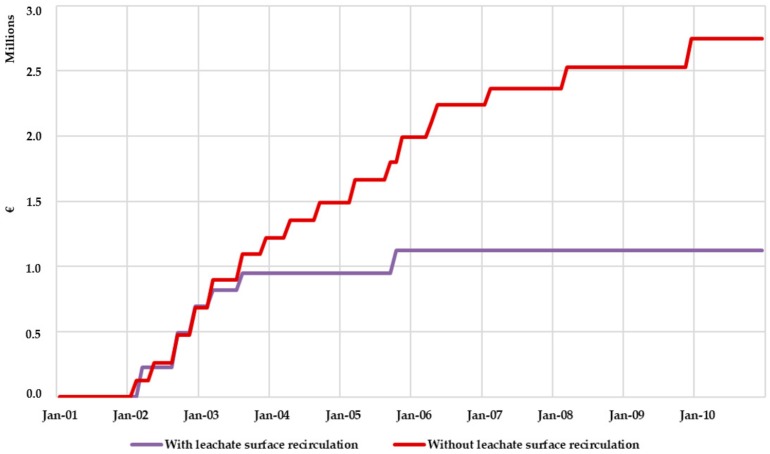
Accumulated cost of leachate treatment in external facilities.

**Figure 23:**
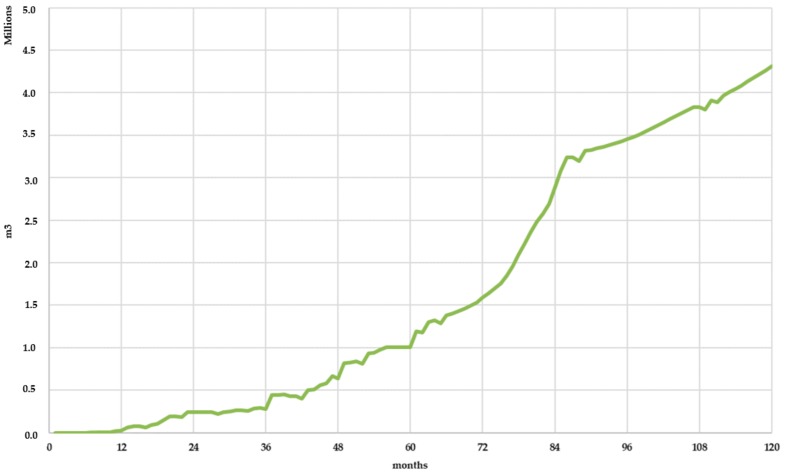
Effect of leachate recirculation on biogas production.

Figure 21, Figure 22 and Figure 23 compare those results obtained by BIOLEACH simulations for scenarios 1 and 2. Figure 21 shows the effect of superficial leachate recirculation on the temporal distribution of leachate that is necessary to transfer to external water treatment facilities. The number of leachate transfers is reduced from 16 (Scenario 1) to 6 (Scenario 2). For Scenario 2 it was also observed that leachate transfer to external facilities are more distanced over time.

Figure 22 shows the comparison of the accumulated cost of external leachate management considering or not surface recirculation. In the first two and a half years the external leachate management costs are similar in both scenarios. However, after month 30, the costs for Scenario 1 continue increasing while for Scenario 2 these costs stabilized until they remained practically constant throughout the rest of the simulation period.

The effect of leachate recirculation also increased biogas production, as the moisture content conditions inside the landfill were closer to the optimal conditions. Figure 23 shows the increase in biogas production due to leachate superficial recirculation. Biogas production increased 3 million m^3^ in the 10 years of simulation due to leachate recirculation techniques (Scenario 2). This value represents an increase of 3.75% compared to the total biogas production for Scenario 1 in the same simulated period.

## 6. Conclusions

This paper introduces BIOLEACH, a new decision support model for the joint evaluation of biogas and leachate productions on MSW bioreactor landfills. BIOLEACH provides an easy-to-use tool that ensures optimal and sustainable environmental management of the landfill. The main characteristics of the BIOLEACH model are: (i) BIOLEACH is a bioreactor management model. It allows for the optimal management of leachate (minimizing the volume of leachate to be managed externally) and biogas (maximizing its production), (ii) the model considers real characteristics of the waste stored in the landfill, (iii) the model simulates the behavior of the landfill as a bioreactor, obtaining the optimal moisture conditions inside the waste mass that allow maximizing biogas production without compromising the stability of the waste slopes, (iv) BIOLEACH has been programmed as a monthly scale model and it can be easily linked to the observations of the weather station located near the landfill. Therefore, the model allows simulating the landfill response to precipitation events of high return period which are usually left unnoticed by annual scale models, (v) the model is flexible and can be adapted to any type of MSW, no matter if it comes from a treatment plant or it is collected house by house, (vi) the user must provide the chemical composition of the MSW distinguishing between rapidly biodegradable waste (RDW), slowly biodegradable waste (SDW) and non-decomposable waste (NDW), and (vii) BIOLEACH allows for a simultaneous control of leachate and biogas productions through a water balance equation approach. The model controls the inputs and outputs of water at each layer in which the landfill has been discretized on a monthly operation basis.

To demonstrate the use of BIOLEACH, the joint evaluation of leachate and biogas production on a real MSW landfill located in Murcia Region (Spain) was simulated. Two different scenarios were designed, considering or not the effect of leachate recirculation over the landfill surface during a ten years period at monthly scale. Results clearly show the impact of recirculation over leachate production and the benefits of using BIOLEACH as a bioreactor management model to support landfills operators.

Further research using BIOLEACH focus on the simulation on real MSW landfills located in semiarid basins using leachate recirculation methods and bioreactor management techniques. Results obtained so far show that BIOLEACH is a suitable tool to perform these analyses.

## Figures and Tables

**Table 1:** Main features of available models for leachate and landfill gas production.

Features	HELP [32]	MODUELO [5,33]	LAST [34]	BIOLEACH (This Work)
Progressive waste disposal	No	Yes	Yes	Yes
Aftercare period	Yes	Yes	Yes	Yes
Landfill discretization	Yes	Yes	Yes	Yes
Vertical flow	Yes	Yes	Yes	Yes
Horizontal flow	Only in drainage layer	Yes	Only in drainage layer	No
Different waste type	Yes	Yes	Yes	Yes
Waste initial moisture	Yes	Yes	Yes	Yes
Actual evapotranspiration	Yes	Yes	Yes	Yes
Water storage capacity	Yes	Yes	Yes	Yes
Waste compression	No	Yes	Yes	Yes
Released water due to compression	No	Yes	Yes	Yes
Waste biodegradation	No	Yes	Yes	Yes
Biogas production	No	Yes	Yes	Yes
Temporal changes of waste properties	No	Yes	Yes	No
Bioreactor management	No	Yes	No	Yes

**Table 2:** MSW characterization of Murcia Region landfill used on the model application.

Type	Component	Total Weight (kg)	Water Content (%)	C	H	O	N	S	Ashes
RDW	Food	17.4	70	48.0	6.4	37.6	2.6	0.4	5.0
RDW	Paper	23.8	50	43.5	6.0	44.0	0.3	0.2	6.0
RDW	60% Garden waste	3.9	60	47.8	6.0	38.0	3.4	0.3	4.5
SDW	40% Garden waste	3.9	30	47.8	6.0	38.0	3.4	0.3	4.5
SDW	Wood	1.1	30	49.5	6.0	42.7	0.2	0.1	1.5
SDW	Textile	22.9	10	55.0	6.6	31.2	4.6	0.2	2.5
NDW	Plastic	17.1	4	-	-	-	-	-	-
NDW	Glass	7.4	2	-	-	-	-	-	-
NDW	Metals	1.8	3	-	-	-	-	-	-
NDW	Others	4.7	30	-	-	-	-	-	-

**Table 3:** List of the bioreactor management model parameters.

Block 1 General Characteristics of Each MSW Layer	Block 2 Water Balance Equation Calculations at the Current Layer	Block 3 Control of the Leachate Volume Stored in Pond before Recirculation	Block 4 Recirculation Criteria	Block 5 Control of the Leachate Volume Stored in Pond after Recirculation
Thickness of MSW layer (m) Thickness of covering layer (m) MSW density (kg/m^3^) Covering material density (kg/m^3^) MSW moisture content (%) Coating layer weight (kg) MSW total weight (kg) MSW water content weight (kg) MSW dry weight (kg) Net infiltration or recirculation (mm) Net infiltration or recirculation weight (kg) Biogas density (kg/m^3^) HPF: High Precipitation Factor (%)	Optimal biogas production (m^3^) Weight of the actual production of biogas (kg) Weight of water consumed in biogas formation (kg) Weight of water vapor produced in biogas formation (kg) Weight of water present in the MSW after biogas formation before possible leaching (kg) Dry weight of MSW after biogas formation (kg) Weight of existing MSW over the centre of each level (kg) Field capacity (%) Maximum weight of water retained by the MSW (kg) Weight of the remaining water up to the field capacity (kg) Weight of water retained in the MSW after biogas formation (kg) Total final weight of the layer (kg) Weight of leachate produced at each level per unit area (kg/m^2^) Surface area of each layer (m^2^) Leachate volume produced by the landfill to be stored in the pond (m^3^)	Volume of leachate stored in the pond (m^3^) Volume of leachate + rainfall stored in the pond (m^3^) Volume of leachate to be managed (volume of leachate + precipitation evaporation) (m^3^)	Efficiency of the biogas production process (available water / water required) (%) Volume of biogas actually produced (m^3^) Maximum biogas production (m^3^) Volume of water that is necessary at each level to achieve optimal biogas production (m^3^) α: target water content inside MSW β: target water content over Field Capacity Volume of water that is needed at each level to reach a percentage (β) of field capacity (m^3^) Recirculation criterion No. 1: volume of water that must be provided in each infiltration trench to guarantee optimal biogas production at the 12 levels below that ditch (m^3^) Recirculation criterion nº2: volume of water that must be provided in each infiltration ditch so that the final humidity of the 12 levels below that ditch is equal to a percentage (β) of the field capacity (m^3^)	Volume of leachate stored in the pond (m^3^) Volume of leachate + rainfall stored in the pond (m^3^) Volume of leachate to be managed (volume of leachate + precipitation - evaporation) (m^3^) Volume of recirculation suggested by BIOLEACH in each infiltration trench (m^3^) Total volume actually recirculated in each of the infiltration ditches as decided by the landfill manager (m^3^) Final volume of leachate stored in the pond after recirculation (m^3^)
